# Beyond the Biological Clock: A Life-Course and Performance Analysis of Masters Swimming World Championships Participation

**DOI:** 10.3390/sports14100430

**Published:** 2026-10-03

**Authors:** Sabrina Demarie, Alessandro Scotto di Palumbo, Flavia Guidotti, Christel Galvani, Veronique L. Billat

**Affiliations:** 1 Department of Movement, Human and Health Sciences, University of Rome “Foro Italico”, 00135 Rome, Italy; a.scottodipalumbo@uniroma4.it; 2 Department of Human Sciences and Promotion of the Quality of Life, San Raffaele Roma Open, 00166 Rome, Italy; flavia.guidotti@uniroma5.it; 3 Exercise & Sport Science Laboratory, Università Cattolica del Sacro Cuore, 20123 Milan, Italy; christel.galvani@unicatt.it; 4 Laboratoire Informatique, Bio-Informatique et Systèmes Complexes (IBISC), EA 4526, Université Paris-Saclay, 91034 Evry, France; veroniquelouisebillat@gmail.com

**Keywords:** aging, masters athletes, sport participation, life course, performance decline, segmented regression, model selection

## Abstract

Age-related declines in sport participation and performance are commonly attributed to physiological constraints. This retrospective observational study compared the age patterns of participation and performance in masters swimming using 26,789 individual-event performances (expressed as the percentage change relative to each subgroup’s mean baseline race time at the youngest age) from three World Aquatics Masters Championships (2023–2025), in four strokes, sprint (50–100 m) and endurance (400–800 m) events, and both sexes. Participation, defined as individual-event performances (not unique swimmers) per million inhabitants of the corresponding age group, was modelled with polynomial and segmented regression; race-time deterioration (% above the 25–29-year baseline) with linear, quadratic and segmented regression compared by the corrected Akaike Information Criterion (AICc). Participation followed an inverted-U trajectory in all sex × distance groups, decreasing from 27 to approximately 37 years, rising to a peak in the early fifties (≈52 years) and declining thereafter. Race times deteriorated monotonically and non-linearly; quadratic and segmented models were statistically equivalent (|ΔAICc| < 2), with the rate of deterioration rising at approximate transition ages (not precise physiological thresholds) between the late fifties and the late sixties depending on the group. This divergence suggests that, up to the sixth decade, the age pattern of participation is not a simple reflection of the age pattern of performance. We propose, as a hypothesis, not tested here and to be examined with individual-level longitudinal data, that life-course and social factors may shape engagement in competitive masters swimming before physiological constraints become predominant.

## 1. Introduction

The suggestion that older people should engage in “active aging” has become a dominant local, national and international policy agenda, primarily aimed at extending the quality of life of older adults [1,2,3]. Institutions worldwide are reformulating policies to encourage the engagement of seniors through accessible programmes that promote well-being and self-sufficiency, and policymakers, practitioners and scholars are urged to work together toward a better old age through an informed understanding of appropriate provision strategies [4,5]. Different life stages can represent critical transition points that affect both the extent and the duration of senior athletes’ participation [6]. Masters swimmers, a self-selected group of people who compete under standardised conditions across several decades of the lifespan, display a marked resistance to the usual consequences of aging and can therefore be regarded as a living laboratory offering a valuable perspective for gerontology [7,8,9]. From a social science and public health perspective, masters sport is a large-scale demonstration of sustained voluntary engagement in vigorous physical activity well into old age. Studies of masters athletes have shown that competitive sport can support identity management, perceived social value and resistance to ageist stereotypes among older adults [1,10,11]. It has also been proposed that the perceived value of elite sport in a country is an important predictor of the subjective well-being of its population, in support of the “virtuous cycle of sport” idea [12]. The visibility of national athletes competing on the world stage, together with the media exposure and public interest generated by such events, may inspire individuals in the participating countries to adopt or maintain an active lifestyle, thereby contributing to a wider culture of lifelong participation in sport and physical activity [13]. Since high-level masters swimmers can be viewed both as an extension of elite performance and as a structural manifestation of active aging among the leisure class, their participation in world championships may encourage participation among athletes from their own nation, while also promoting broader public engagement in physical activity.

Unlike most aging research, which studies people as they become sedentary, research on masters swimming studies people who actively oppose that trajectory [14]. The joint analysis of performance and participation trends may help to discern the extent to which participation across age groups is driven predominantly by physiological determinants or also by broader socio-structural forces [15,16]. The physiological explanation assumes that, as athletes age, the progressive loss of muscle mass, cardiovascular efficiency and neuromuscular coordination makes sustained competitive participation increasingly difficult, ultimately causing dropout from the sport [17,18,19]. Cross-sectional and longitudinal analyses of masters swimming records have consistently shown a slow, near-linear decline in performance from the thirties to the sixth or seventh decade of life, followed by an accelerated, non-linear deterioration beyond that age [20,21,22]. Whether this acceleration is better described as a smooth, progressively steepening process or as a discrete change in the rate of decline is, however, an open methodological question, and the answer determines whether a single “transition age” can meaningfully be identified; segmented regression has recently been used to locate such age-related breakpoints in masters swimming [23]. If physiology were the primary driver of participation, the decline in participation would be expected to parallel the decline in performance and to vary systematically across events and sexes: both declines should be steeper in sprint events, which demand explosive power, more gradual in endurance events, which rely on aerobic efficiency, and potentially divergent between sexes [24,25,26,27,28]. Such a pattern would be consistent with the progressive decrease in participation in physical activity and sport with advancing age that is commonly reported in different countries [29,30,31,32]. On the other hand, individuals do not age in a biological vacuum: their behaviours and athletic trajectories are synchronised with a culturally constructed “social clock” [3,33]. If the age pattern of participation does not parallel that of performance and is stable across distances and sexes, a social mechanism could be responsible for the age gradient in participation. A sociological interpretation would suggest that participation in sport and physical activity is shaped by cultural norms about aging and athleticism, shifting life-course obligations, institutional barriers and the social meaning ascribed to competitive sport at different ages [4,34]. The approach used to examine work–family conflict, which seeks to integrate individual and social constraints, could thus be applied to sport participation [35,36]. Distinguishing between these explanations has substantial implications for how sporting bodies, public health practitioners and policymakers design interventions aimed at sustaining athletic engagement into later life [2,37]. Evidence on the pattern of sport participation across the aging process could guide the investigation of the moderating factors that should be considered when developing programmes to promote active aging in media reports, government policies, and sport and exercise management [8,38].

The aim of the present study was therefore to examine whether participation in competitive masters swimming follows the same age pattern as performance, which would be consistent with a physiologically limited competitive career, or whether it follows a different pattern, compatible with a hypothesised life-course process in which social constraints may shape entry into, exit from and re-entry into competition. To this end, participation and performance at three consecutive World Aquatics Masters Championships were modelled within a single dataset. Participation was operationalised as the number of individual-event performances per million inhabitants of the corresponding age group, and not as the number of unique swimmers. Because both participation and performance were analysed as repeated cross-sections of age groups, the study describes age-related differences at the population level and does not test individual-level mechanisms; the life-course interpretation is therefore proposed as a hypothesis to be tested in future longitudinal work with individual-level socioeconomic, family and motivational data.

## 2. Materials and Methods

### 2.1. Study Design and Data Sources

This retrospective observational study used publicly available competition results. Data were extracted for the World Aquatics Masters Championships held in Kyushu (Japan, 2023), Doha (Qatar, 2024) and Singapore (2025) from the official World Aquatics website (https://www.worldaquatics.com; consulted from 1 September 2025 to 31 March 2026). The variables analysed were the number of individual-event performances (race entries) and the corresponding race times in the individual events of the four strokes (freestyle, backstroke, breaststroke and butterfly) over all race distances (50–800 m). To differentiate between events with markedly different metabolic profiles, the 50 and 100 m events were pooled as sprint events and the 400 and 800 m events as endurance events. The 200 m events were retained only in the analysis of the overall participation counts. Population figures for each country, age group and sex in the three championship years were extracted from the World Bank (https://data.worldbank.org) and United Nations (https://www.un.org) databases (both consulted between 1 and 30 April 2026).

### 2.2. Participants, Performances and Unit of Analysis

The initial dataset, before the application of the exclusion criteria (Section 2.3), comprised 26,893 individual-event performances (men, 14,761; women, 12,132) from 101 countries across six continents: Africa (*n* = 550), Asia (*n* = 10,074), Europe (*n* = 10,608), North America (*n* = 2693), South America (*n* = 1222) and Oceania (*n* = 1746). These performances were achieved by 10,142 unique swimmers; 17.6% of swimmers took part in more than one of the three championships, and swimmers entered a mean of 3.13 events (range 1–16) per championship. Because the same swimmer could contribute several performances within and across championships, individual records were not treated as independent observations: all models were fitted to age-group aggregates (Section 2.5), and the number of performances was used to define the minimum group size required for inclusion, as the numerator of the participation rate (Section 2.5.2) and as the weight in the weighted least-squares analyses. Participation in this study therefore refers to performance-based participation, i.e., the number of individual-event performances, not the number of unique swimmers (Section 2.5.1). Swimmers ranged in age from 25 to 99 years and were grouped in the standard five-year masters categories of the World Aquatics rules.

### 2.3. Inclusion and Exclusion Criteria

To ensure that only valid results of swimmers who completed the full competition were analysed, and to avoid the confounding effects of mixed-stroke and relay events, the following criteria were adopted.

#### 2.3.1. Inclusion Criteria

• performances in individual events;
• performances in single-stroke events;
• performances of swimmers who completed the race.

#### 2.3.2. Exclusion Criteria

• disqualification by the officials for rule violations or any other reason;
• swimmers who did not complete the race;
• age groups with fewer than 30 performances in a given event, a threshold adopted to obtain stable age-group means for regression.

Application of these criteria led to the exclusion of all swimmers in the 90–94 and 95–99 age groups (104 performances), so that the 26,893 performances of the initial dataset were reduced to the 26,789 performances retained for analysis in 13 age groups (25–29 to 85–89 years). All analyses reported below refer to these 26,789 performances.

### 2.4. Ethical Considerations

Competition data were obtained from the official World Aquatics website, where they are made publicly available as part of the federation’s official competition results. All athletes consent to the collection, processing and public dissemination of their competition data during the registration process, in accordance with the applicable World Aquatics regulations and privacy policy. The dataset used in this study consisted exclusively of publicly accessible, de-identified competition results and did not include any personal or sensitive information beyond that already available in the public domain. Accordingly, the study involved the secondary analysis of publicly available data, did not require direct participant involvement or intervention, and was conducted in accordance with the principles of the Declaration of Helsinki. Informed consent for participation in this study was not required under the applicable institutional guidelines. Formal ethical approval was granted as part of a broader research project (University of Rome Foro Italico, CAR 182/2024).

### 2.5. Statistical Analysis

#### 2.5.1. Terminology

The following terms are used consistently throughout the manuscript. *Participation* (participation rate) always refers to performance-based participation, i.e., the number of individual-event performances (race entries with a valid result) per million inhabitants of the corresponding age group and sex, and not the number of unique swimmers. Because a swimmer who enters several events or several championships contributes several performances, participation as defined here reflects both how many swimmers compete and how many events each of them enters; the two components cannot be separated with the available data. The *peak participation age* is the age at which the fitted participation curve reaches its maximum. The *participation breakpoints* are the ages at which a two-breakpoint segmented model of participation changes slope. The *transition age* is the breakpoint of a one-breakpoint segmented model of race-time deterioration, i.e., the estimated age at which the annual rate of deterioration changes; it is reported for every group, together with the model comparison indicating whether a discrete change in rate or a smooth (quadratic) acceleration describes the data better. The transition age is an approximate statistical descriptor, with a resolution of one five-year age group; it is not a precise physiological threshold and does not identify the age at which physiological decline begins. The *slope change* is the difference between the post- and pre-transition slopes of the segmented model (% · yr^−1^). For the quadratic model, the *acceleration* is the second derivative of the fitted curve (2a_2_, % · yr^−2^), which is constant across age. The terms “vertex” and “inflection point” are not used, because the vertex of a parabola and the breakpoint of a segmented model are not equivalent quantities.

#### 2.5.2. Participation

Participation was expressed as the number of performances per million inhabitants of the corresponding age group and sex in the swimmers’ countries for the relevant championship year (Participation Rate (age group) = [Σ Performances (age group)]/[Σ Population (age group)/1,000,000], where Σ Performances (age group) is the sum of performance counts across all race entries, countries, and sexes recorded for that age group, and Σ Population (age group) is the sum of the corresponding single-year, sex-specific population for that age). Each individual-event performance was counted once, so that a swimmer who entered several events contributed several performances; participation therefore denotes performance-based participation (Section 2.5.1) and not a count of unique swimmers. Participation rates were averaged across the four strokes for each five-year age group, and age groups were represented by their midpoints (27, 32, …, 87 years) to obtain a continuous age variable, yielding 13 data points per trajectory.

Participation trajectories were modelled with a two-step approach combining polynomial and segmented regression. First, candidate polynomial models (first to fifth degree) were fitted by ordinary least squares. Model selection was based on the corrected Akaike Information Criterion (AICc), which was preferred to the coefficient of determination (R^2^) because it balances goodness of fit against model complexity and protects against overfitting in small samples [39,40]; the complete AICc comparison is reported in Table 1. The polynomial with the lowest AICc was retained to describe the overall trajectory. The peak participation age was then determined analytically from this polynomial: stationary points were identified by solving its first derivative, only real solutions within the observed age range were retained, the second derivative was used to confirm local maxima, and the domain boundaries were also evaluated as candidates for the global maximum. Second, to locate age-related transitions, a continuous two-breakpoint segmented linear model was fitted. Candidate breakpoint pairs were evaluated by exhaustive grid search over all admissible interior age-group midpoints; for each pair, the parameters were estimated by ordinary least squares and the pair yielding the minimum residual sum of squares was retained. The peak age of the polynomial was compared with the breakpoints of the segmented model as a consistency check between the two complementary approaches.

Because polynomials of high degree fitted to 13 points may be unstable, particularly near the boundaries of the age range where the number of performances and the population denominators are smallest, the robustness of the peak participation age was examined in sensitivity analyses: (i) with the third-degree polynomial; (ii) after exclusion of the two oldest age groups (80–89 years); (iii) with weighted least squares, using the number of performances in each age group as weights; and (iv) with a non-parametric locally weighted regression (LOESS, span = 0.75) [41]. The peak participation age is reported together with the range obtained across these specifications. Given the five-year resolution of the age categories, peak ages and breakpoints are interpreted as approximate values (±one age group) rather than as precise thresholds. The fitted curves were not interpreted beyond 82 years, where the terminal behaviour of the polynomials is driven by very small counts and population denominators.

#### 2.5.3. Race Times

Because absolute race times cannot be compared between sexes or distances, race-time deterioration was expressed as the percentage change relative to a baseline (T_0_), which provides a dimensionless, sex- and distance-neutral metric. For each stroke × distance × sex subgroup, T_0_ was the mean race time of the 25–29-year age group (midpoint 27 years, the youngest group with ≥30 performances), and the relative deterioration of each age group was computed as

Δ%(age) = [T(age) − T_0_]/T_0_ × 100,

where T(age) is the mean race time of the age group; a positive Δ% indicates that the age group is slower than the baseline.

For each subgroup the three championships data were pooled together before fitting and three models were fitted by ordinary least squares to the 13 age-group values of Δ%: a linear model, a quadratic model (Δ% = a_0_ + a_1_·age + a_2_·age^2^) and a one-breakpoint segmented linear model with two segments joined at an estimated breakpoint [42]. The breakpoint was estimated as the value minimising the residual sum of squares over the age-group midpoints, i.e., with a five-year resolution, and its 95% confidence interval was obtained by bootstrap resampling of the age-group means. The existence of a change in slope was tested with Davies’ test [43]. Models were compared with AICc; ΔAICc was computed as AICc (alternative model) − AICc (segmented model), so that positive values favour the segmented model. Following common practice, |ΔAICc| < 2 was interpreted as equivalent support for the two models and |ΔAICc| > 10 as essentially no support for the model with the higher AICc [40].

In sprint events, four strokes were available for each sex. Because averaged confidence limits do not constitute a confidence interval for a pooled estimate, stroke-specific statistics were not averaged; instead, a single model per sex was fitted to the stroke-specific Δ% series pooled across strokes (4 strokes × 13 age groups = 52 points), which yelds a porper confidence interval for the pooled transition age. Endurance events (400 and 800 m) were contested in freestyle only, so that each sex group comprised a single series. Because the models were fitted to age-group means, the reported R^2^ values quantify the proportion of variance among the 13 age-group means explained by age, not the variance among individual performances [44]. Since age is constant within an age group, a weighted least-squares fit of the age-group means with weights equal to the number of performances yields the same parameter estimates as an ordinary least-squares fit of the individual performances; to verify that aggregation did not affect the conclusions, all models were therefore also refitted by weighted least squares.

The transition age, the slopes before and after it and the slope change were taken from the segmented model in all groups. When the quadratic model was equally or better supported, the quadratic acceleration (2a_2_) was reported in addition, and the transition age was interpreted as the approximate centre of the age range over which the rate of deterioration increases rather than as a discrete or precise physiological threshold; in all groups it is an approximate value (±one five-year age group). For graphical purposes, the fitted curves were displayed on a one-year age grid.

Analyses were conducted in Python 3.11 with NumPy 1.26.4, Pandas 2.2.2, Plotly 5.22.0, SciPy 1.18.1 and Statsmodels 0.15.0 (segmented, weighted least-squares and LOESS fits). Statistical significance was set at *p* < 0.05.

## 3. Results

### 3.1. Overall Age-Related Participation Trajectory

Table 1 reports the AICc comparison of the polynomial models. The fourth-degree polynomial had the lowest AICc (136.1; R^2^ = 0.93, adjusted R^2^ = 0.88). The two-breakpoint segmented model located the participation breakpoints at 37 and 52 years, in agreement with the peak participation age of 52 years derived from the polynomial. In the sensitivity analyses, the estimated peak participation age ranged from 43 to 55 years. The third-degree polynomial yielded an earlier peak at 43 years, whereas the other sensitivity specifications produced estimates between 52 and 53 years. Thus, although most specifications supported a peak in the early fifties, the cubic model indicated some sensitivity to model choice.

Figure 1 shows the overall trajectory (all distances, 200 m included, and both sexes). Participation decreased from the youngest age group (25–29 years) to approximately 37 years, increased to a peak at approximately 52 years and decreased progressively thereafter, into the eighties. The apparent upturn of the fitted curve in the oldest age groups is a boundary artefact of the polynomial (Section 2.5.2) and was not interpreted.

### 3.2. Sprint Versus Endurance Participation by Sex

As shown in Figure 2, all four sex × distance groups displayed the same inverted-U shape, with peak ages between 53 and 59 years. Among men, sprint participation peaked at 54 years and reached the highest rate of any group (approximately 17.3 performances per million inhabitants), whereas endurance participation peaked at 55 years at a substantially lower rate (approximately 10.4 per million). Among women, sprint participation peaked at 53 years (approximately 12.6 per million) and endurance participation at 59 years, with the lowest rate of the four groups (approximately 7.6 per million). Beyond the peak, sprint participation declined steeply in both sexes to its lowest values at 75–80 years, whereas endurance participation declined more gradually. The upturn of the men’s endurance curve after approximately 78 years, which appears to overtake sprint participation in the mid-eighties, is based on age groups containing about 30 performances divided by small population denominators and coincides with the terminal segment of a fourth-degree polynomial; it was therefore treated as a statistical artefact and not as evidence of a late-life shift toward endurance events (Section 4.9).

### 3.3. Race-Time Deterioration: Model Comparison and Transition Ages

Table 2 summarises the race-time models. In all four groups, both the quadratic and the segmented model fitted the age-group means far better than the linear model (ΔAICc of the segmented model versus the linear model between 15.4 and 25.1; R^2^ 0.95–0.99 versus 0.82–0.94), demonstrating that deterioration accelerates with age. In contrast, the segmented and quadratic models were statistically indistinguishable: |ΔAICc| was below 2 in every group (−1.02, −0.24, +1.18 and −1.81 for female sprint, male sprint, female endurance and male endurance, respectively), and the segmented model was preferred in half of the stroke-specific sprint fits in each sex. The data therefore support a non-linear, progressively accelerating deterioration but do not discriminate between a smooth acceleration and a discrete change in rate. Davies’ test rejected the hypothesis of no change in slope in all groups (*p* < 0.001 in every stroke-specific fit.

The approximate transition ages estimated by the segmented model were 62 years (female sprint), 67 years (male sprint), 67 years (female endurance) and 57 years (male endurance) (Table 2). Given the five-year resolution of the age categories and the fact that the confidence intervals of the two sexes adjoin or overlap within each distance, the sex differences in transition age (one age group in sprint events, two in endurance events) should be regarded as indicative. The transition ages themselves should likewise be read as approximate values (± one age group), not as precise physiological thresholds. Before the transition, race times worsened by 0.6–1.2% per year; after it, by 2.0–3.7% per year, i.e., 2.4- to 5.0-fold faster (Table 2). Post-transition slopes were steeper in sprint (3.4–3.7% · yr^−1^) than in endurance events (2.0–2.9% · yr^−1^). In endurance events, the later transition age of women (67 versus 57 years) was accompanied by a pre-transition slope twice as steep as that of men (1.20 versus 0.60% · yr^−1^) and by a steeper post-transition slope (2.92 versus 2.01% · yr^−1^): women’s endurance times deteriorated more uniformly across the age range, whereas men’s deterioration was slower until the late fifties and accelerated thereafter. The later transition age in women therefore reflects a more nearly linear trajectory rather than a longer preservation of endurance performance.

### 3.4. Relative Race-Time Deterioration with Age

Figure 3 shows the relative race-time deterioration (Δ%) of the four groups together with the fitted curves. In sprint events, both sexes remained within approximately 10% of the baseline until the mid-forties, after which deterioration accelerated progressively, reaching approximately 100% above the baseline by 87 years in both sexes; the women’s approximate transition age was one age group earlier than the men’s (62 versus 67 years), with a comparable magnitude of deterioration by the mid-eighties. In endurance events, the sexes diverged: men’s times deteriorated slowly until the late fifties and more rapidly thereafter, reaching approximately 75–80% above the baseline by 87 years, whereas women’s times deteriorated more uniformly across the age range, were more deteriorated than men’s at every age group from the late thirties onward, and exceeded 100% above the baseline by 87 years.

## 4. Discussion

### 4.1. Main Findings

This study compared, within a single dataset of three World Aquatics Masters Championships, the age pattern of participation (measured as performances per million inhabitants, not as unique swimmers) with the age pattern of performance. Participation followed an inverted-U trajectory in both sexes and in both sprint and endurance events: it decreased from the late twenties to approximately 37 years, increased to a peak in the early fifties and declined thereafter. Performance, in contrast, deteriorated monotonically and non-linearly, with the rate of deterioration rising at approximate transition ages between the late fifties and the late sixties depending on the group (not precise physiological thresholds). The two trajectories therefore diverge over most of the age range: participation increases between approximately 37 and 52 years while performance is already deteriorating, and participation begins to decline about a decade before the acceleration of performance deterioration. The inverted-U pattern differs from the monotonic decrease in sport and physical-activity participation with age that is commonly reported in population surveys [3,29,30,31,32], and its similarity across sexes and physiologically heterogeneous events suggests that, up to the sixth decade, the age pattern of participation in competitive masters swimming is not a simple reflection of the age pattern of performance.

### 4.2. What the Participation Trajectory Can and Cannot Show

The design imposes clear limits on interpretation. The sample is a repeated cross-section of age groups drawn from three championships, not a cohort followed over time. The trajectory therefore describes differences in participation between age groups at a given time, not the life course of any individual swimmer: a higher participation rate at 50 than at 35 years does not demonstrate that swimmers who left the sport in their thirties returned to competition in their forties, since the same pattern could arise from age-specific propensities to compete that differ between birth cohorts, or from later entry into the sport. In addition, participation was measured as individual-event performances per million inhabitants and not as unique swimmers; differences between age groups may therefore reflect the number of swimmers who compete, the number of events that each swimmer enters, or both, and the two components cannot be separated with the available data. Moreover, personal motivation, family situation, employment, income and retirement were not measured, so that none of the socioeconomic or motivational mechanisms discussed below could be tested. The interpretive framework proposed below should therefore be read as a set of hypotheses that are compatible with the observed pattern and with the literature, and that could be tailored to the cultural setting of each nation [45,46,47], not as an explanation established by the present data.

### 4.3. A Life-Course Hypothesis for the Three Phases

The interpretations proposed in this section are hypotheses generated from the timing of the three phases and from the literature: the dataset is cross-sectional and contains no individual-level socioeconomic, family, employment or motivational variables, so none of the mechanisms below were measured or tested. With these caveats, the three phases of the participation trajectory are consistent with life-course accounts in which sport engagement is regulated by social, economic and institutional circumstances that change with age [33,47]. The lower participation of the youngest age groups (27–37 years) could reflect exit from, or postponement of, demanding competitive activity during a life stage characterised by early-career consolidation, partnership formation and parenthood, which are reported to monopolise discretionary time and energy [35,48,49]. The higher participation observed between approximately 37 and 52 years could correspond to a socially patterned release of these obligations, with more discretionary time and financial resources and a cultural shift toward active aging [33]; qualitative studies of masters swimmers describe motives such as identity, social bonding and re-engagement after family or career transitions [8,50], and the peak in the early fifties coincides with the age range in which life events such as children leaving home and, in some countries, early retirement become frequent [51,52,53]. Whether these events applied to the swimmers studied is unknown. Because attending a world championship, as opposed to a local or national event, requires substantial travel costs and time off work [54], the rise in participation may also reflect increased disposable income and discretionary time [37]. Finally, the post-peak decline after approximately 52 years may reflect the combined influence of biological attrition, consistent with the performance deterioration documented here, and structural barriers to continued competitive travel and training at advanced ages, including health, financial and logistic constraints that the dataset does not capture [21,55]. Each of these mechanisms is testable with individual-level data, but none was tested here.

### 4.4. Alternative Explanations

Other processes could generate a similar inverted-U pattern and merit consideration. First, participants at world championships are a strongly self-selected population: athletes who remain competitive into their sixties and seventies have, by definition, avoided or overcome the health conditions that remove most of their peers from sport [7]. This survivorship effect could shape the descending limb of the curve independently of any social mechanism. Second, the three championships were held between 2023 and 2025, so that the age groups correspond to birth cohorts ranging from the 1930s to the late 1990s, whose exposure to organised swimming in youth, retirement ages and family structures differed substantially; part of the pattern may therefore be a cohort effect rather than an age effect. Third, all three championships were held in Asia, and travel distance and cost may have affected the age composition of the participants from different continents. Fourth, the models are based on a small number of age groups (13) with five-year categories, so that the phase boundaries are approximate. Fifth, participation was measured as performances rather than unique swimmers, so that age differences in the number of events entered per swimmer could also contribute to the pattern. Distinguishing between these accounts would require data linking individual participants across editions and to their employment, family and financial circumstances, which were not available.

### 4.5. Performance Deterioration: A Progressively Accelerating Decline

Unlike participation, performance deteriorated in all groups from the youngest age group onward, slowly at first and then more rapidly. Both the quadratic and the segmented model described the age-group means far better than a linear model, confirming that the deterioration accelerates with age, in agreement with previous cross-sectional and longitudinal studies of masters swimmers [9,17,18,20,21,22,56]. However, the two non-linear models were statistically equivalent in every group (|ΔAICc| < 2). The present data therefore do not establish whether deterioration proceeds through a discrete change in rate, as implied by the term “biphasic”, or through a smooth acceleration; with 13 age-group means, the two descriptions are not distinguishable. Although Davies’ test provided evidence against a constant slope within the segmented-regression framework, it did not establish that the underlying age-related deterioration follows a discrete transition rather than a smooth acceleration. The AICc comparisons did not discriminate between these two descriptions. Accordingly, the transition ages reported in Table 2 (57–67 years) should be interpreted as the approximate centre of the age range over which the rate of deterioration increases, with an uncertainty of at least one five-year age group, rather than as precise physiological thresholds. Within this limit, the ages at which the rate increased are consistent with the acceleration of decline in the sixth and seventh decades reported for swimming performance [20,21,22] and with the cumulative losses in aerobic capacity, muscle mass, neuromuscular function and recovery capacity that become increasingly difficult to compensate in later life [17,18,19].

### 4.6. Sprint and Endurance Events and Sex Differences

In sprint events, participation peaked at 53–54 years in both sexes, about a decade before the approximate transition age of performance deterioration (approximately 62 and 67 years in women and men, respectively). The descending limb of participation therefore begins while sprint performance is still deteriorating slowly, which is difficult to reconcile with the view that engagement in sprint competition is governed by the preservation of explosive capacity alone. The sex difference in the sprint transition age amounts to one age group, with overlapping confidence intervals, and should not be over-interpreted.

In endurance events, women’s participation peaked latest of all groups (59 versus 55 years in men) and women’s approximate transition age was later than men’s (67 versus 57 years). At first sight, this might suggest that women preserve endurance performance a decade longer than men. The slopes in Table 2 do not support this reading: women’s endurance times deteriorated twice as fast as men’s before the transition (1.20 versus 0.60% · yr^−1^), were more deteriorated than men’s at every age group from the late thirties onward (Figure 3), and continued to deteriorate faster after the transition (2.92 versus 2.01% · yr^−1^). The later transition age in women therefore reflects a more nearly linear trajectory, in which the change in rate is less marked, rather than a longer period of preserved performance. This observation is compatible with reports that the sex difference in endurance performance widens with advancing age [57], whereas a narrowing or reversal of the sex gap has been described in some endurance events and in age-group records [24,25,58,59,60,61]; the two sets of findings are not contradictory, because mean performances of all finishers and record performances describe different populations.

### 4.7. Interpreting the Sex Difference in Endurance Events

The determinants of the sex difference in the endurance trajectories could not be identified from the present data, which contain no physiological, hormonal, psychological or socioeconomic measurements. Several hypotheses may be advanced for future testing. The menopausal transition, which typically occurs between 45 and 55 years, is associated with hormonal changes linked to loss of lean mass and bone mineral density, sleep disruption, mood disturbances and increased cardiovascular risk [62,63,64,65,66,67], and menopausal symptoms are reported by female endurance athletes to affect training and performance [68,69,70,71,72]; the steeper deterioration of women’s endurance times in mid-life is compatible with, but does not demonstrate, such an influence, and the timing of menopause relative to the age groups studied could be examined in future work with individual data [73]. Social and motivational factors may also contribute: older women’s sport participation remains strongly shaped by gender norms, although older women place greater value than older men on the health benefits of sport [3,74]; qualitative studies report that women who continue competing adopt a resolute “no excuses” orientation and draw on personal responsibility for health and on family support [16,75,76]; and international masters competition is associated with high socioeconomic capital [77]. Whether such factors influence the endurance trajectory of women differently from that of men cannot be determined here. The comparison between the sexes should also take into account the difference in participation, because the smaller number of women competing in endurance events at advanced ages may be differently selected than men [57]. We therefore refrain from interpreting the transition age of 67 years as a threshold at which physiological aging overrides behavioural or social buffers, an interpretation that would go beyond the data.

### 4.8. Dissociation Between Participation and Performance

Participation rises toward the early fifties even though race times have already begun to deteriorate. While not an observed result, the proposed influence of life-course circumstances is a hypothesis that follows from this observation. Taken together, the invariance of the inverted-U participation trajectory across physiologically heterogeneous events and both sexes, and its divergence from the monotonic race-time trajectories, are consistent with the hypothesis that, up to the sixth decade of life, factors other than physiological capacity may play a larger role in the decision to compete in world-level masters swimming, whereas the race-time trajectories suggest the approximate stage of aging at which physiological constraints become the principal determinant of performance among those who continue to compete, without identifying a precise physiological threshold. The design does not allow the nature of these factors to be established, and the data are equally compatible with the cohort and selection processes discussed in Section 4.4.

### 4.9. Limitations

Several limitations should be considered when interpreting these findings. First, the cross-sectional design does not follow individual swimmers over time; the curves describe the age composition of the championships, not individual trajectories, and are vulnerable to cohort and survivorship effects. Second, the sample is restricted to athletes who attended world championships, the most competitive and best-resourced tier of masters swimming; the inverted-U participation trajectory may therefore reflect structural privilege rather than a general feature of aging, and the patterns among recreational or nationally competing masters swimmers may differ substantially. Third, no individual data on employment, family structure, income, health or motivation were available; the mediators proposed in Section 4.3 were inferred from the timing of the phases and from the literature, not measured, and therefore remain hypotheses. Fourth, all models were fitted to age-group aggregates: the high R^2^ values quantify the fit to 13 age-group means and not the variance among individual performances, the resolution of transition ages and breakpoints is limited by the five-year age categories, and repeated participation by the same swimmers within and across championships means that the number of performances overstates the number of independent observations. Moreover, participation was measured as performances rather than unique swimmers, so that the data cannot distinguish differences in the number of swimmers from differences in the number of events entered per swimmer. Fifth, the quadratic and segmented models of race times were statistically equivalent, so that the identification of a discrete transition age is a modelling convention rather than a demonstrated feature of the data. The transition ages are accordingly approximate (five-year resolution) and do not represent precise physiological thresholds. Sixth, the fitted participation curves are unstable at the upper boundary of the age range, where counts and population denominators are small, so that the apparent late-life upturn in men’s endurance participation should not be interpreted. Finally, the dataset spans only three championship editions (2023–2025), all held in Asia, and a single governing body’s competition structure, which may limit generalisability to other sports, regions and periods.

## 5. Conclusions

The present findings do not establish causal mechanisms, but they show that the age pattern of participation (performances per million inhabitants) in world-level masters swimming differs qualitatively from the age pattern of performance: participation rises to a peak in the early fifties while race times are already deteriorating, and it declines about a decade before the acceleration of performance deterioration. This dissociation is compatible with a hypothesised life-course account, not tested here, in which social and motivational transitions are associated with engagement in competitive sport up to late mid-life, whereas physiological constraints become progressively more important in the later decades. The pattern may also partly reflect cohort-specific circumstances and selection into world championships, and the transition ages of performance deterioration are approximate and should not be interpreted as precise physiological thresholds. Confirmation in longitudinal or successive-cohort designs, at different competitive levels and in other sports, and with individual-level measures of retirement, family status, health and motivation, is required before the three-phase model can be regarded as established.

### Practical Implications

If confirmed, the results would be relevant to the design of interventions targeting active aging. World championships may encourage national participation in competitive masters sport and enhance public engagement in physical activity, and stakeholders could draw on evidence from elite masters sport to inform governance actions [13,78]. Masters performers are exposed to a wide spectrum of organisational stressors, and sport organisations need to remain aware of the social and organisational context they shape around their athletes [54]; reducing the perceived cost of remaining in, or returning to, competitive sport is one way in which federations and support structures can invest in athlete welfare. The lower participation observed between approximately 27 and 37 years, at ages when performance is not yet strongly compromised, generates the hypothesis that retention and recruitment efforts concentrated on older masters swimmers intervene later than the age at which the largest potential gains could be obtained. Whether addressing structural barriers in early adulthood (career demands, childcare responsibilities and time scarcity), together with conditions that may support later engagement (flexible training schedules, social club structures and low-barrier re-entry pathways), increases lifelong competitive engagement is a question for future intervention research with longitudinal follow-up, rather than a conclusion of the present study.

## Figures and Tables

**Figure 1:**
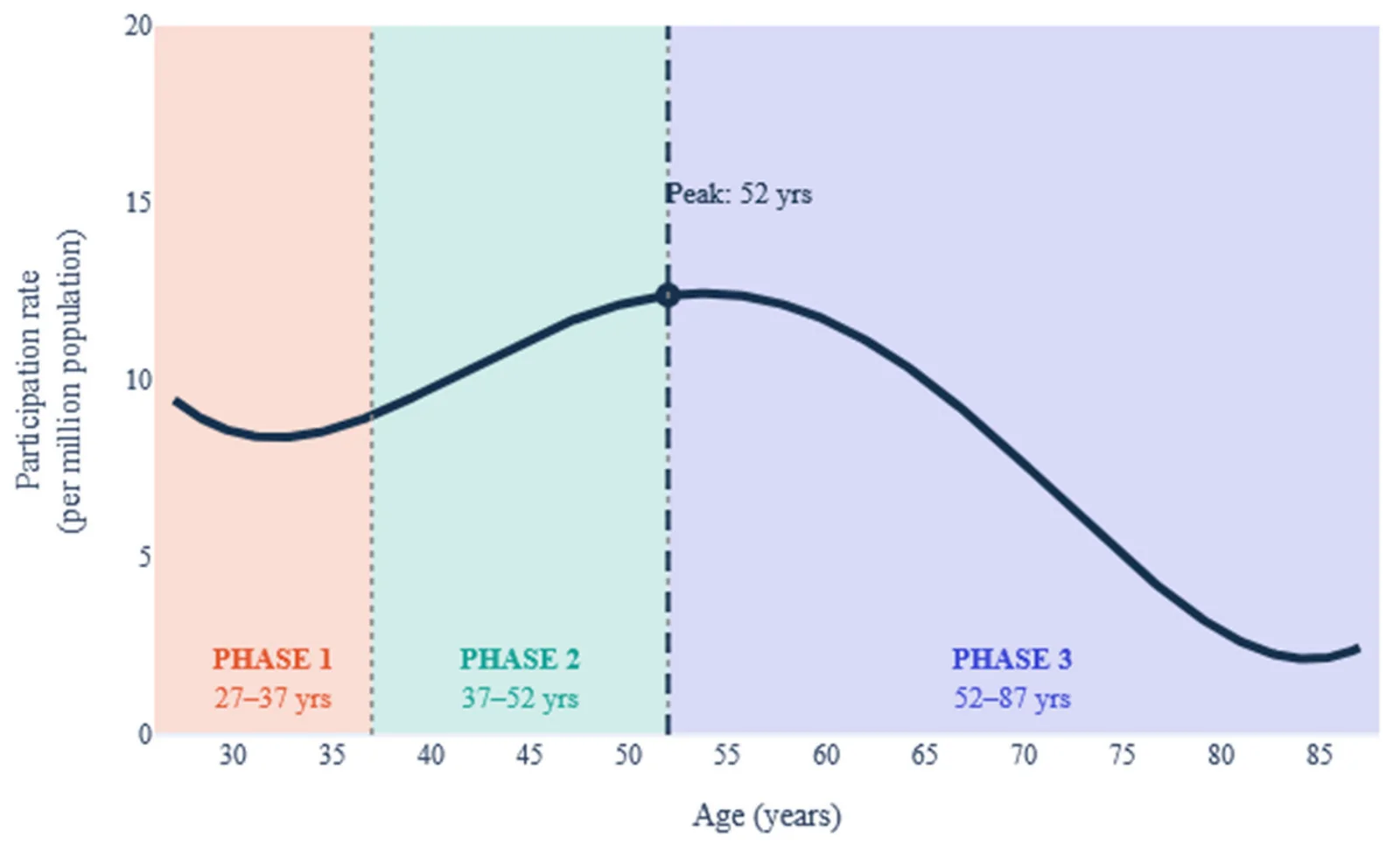
Fitted trajectory of masters swimming participation (performances per million inhabitants of the corresponding age group) versus age at three World Aquatics Masters Championships (2023–2025), all strokes and distances, both sexes. The shaded areas correspond to the three phases described in the text: decline (27–37 years), rise to the peak (37–52 years) and post-peak decline (52–87 years). Phase boundaries are approximate (five-year age categories).

**Figure 2:**
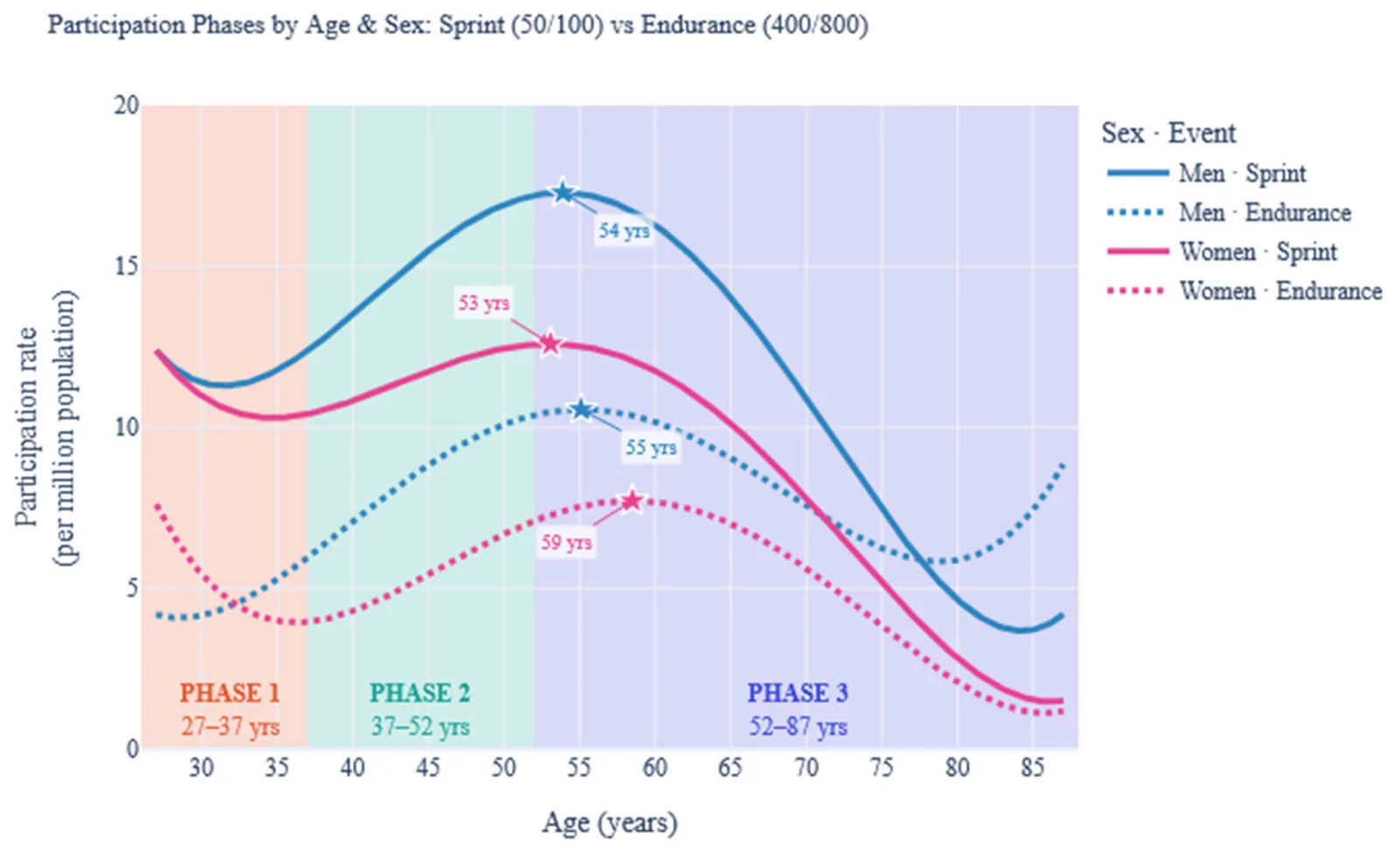
Fitted participation trajectories by age and sex for sprint (50 and 100 m) and endurance (400 and 800 m) events (performances per million inhabitants of the corresponding age group and sex). Stars indicate the peak participation age of each group. The behaviour of the curves beyond approximately 80 years is not interpreted (small counts and denominators; see Section 2.5.2).

**Figure 3:**
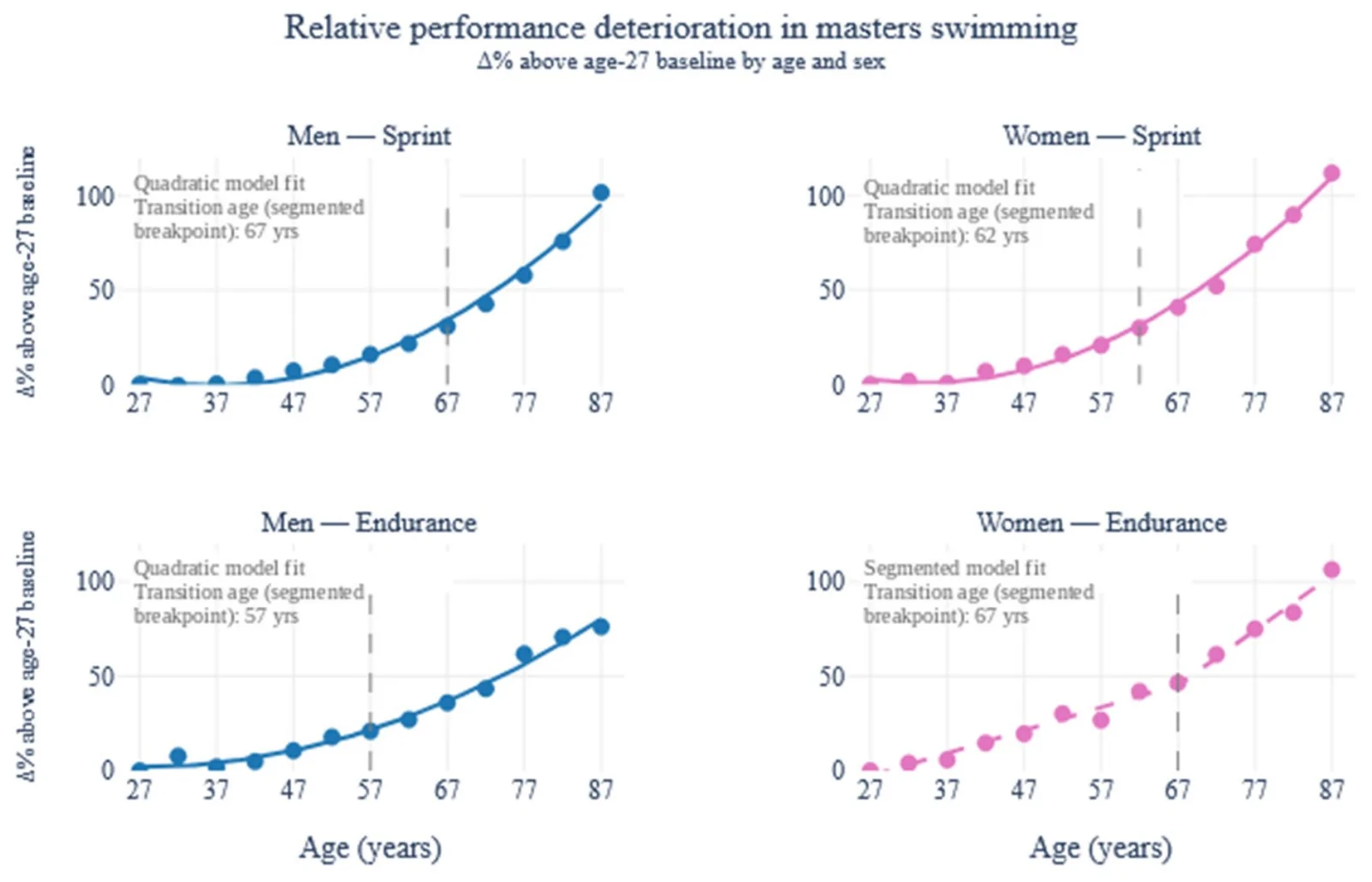
Relative race-time deterioration (Δ%, mean race time of each age group relative to the 25–29-year baseline) versus age in sprint (50 and 100 m) and endurance (400 and 800 m) events for men and women. Points: age-group means (sprint: mean of the four strokes); lines: fitted model with the lower AICc (solid, quadratic; dashed, segmented); dashed vertical lines: approximate transition age (segmented breakpoint; five-year resolution) estimated by the segmented model. In all groups the quadratic and segmented models were statistically equivalent (|ΔAICc| < 2; Table 2).

**Table 1:** Comparison of polynomial models of participation rate (performances per million inhabitants) versus age (13 five-year age groups, 27–87 years).

Polynomial Degree	k	R^2^	Adjusted R^2^	AICc	ΔAICc
1 (linear)	2	0.62	0.58	148.4	12.3
2 (quadratic)	3	0.84	0.81	140.7	4.6
3 (cubic)	4	0.84	0.79	144.9	8.8
4 (quartic)	5	0.93	0.88	136.1	0.0
5 (quintic)	6	0.95	0.91	143.5	7.4

k: number of estimated parameters (including the intercept); AICc: corrected Akaike Information Criterion; ΔAICc: difference from the lowest AICc. The fourth-degree polynomial was retained.

**Table 2:** Race-time deterioration models by sex and distance: model comparison, transition age, and slopes before and after the transition (Δ% of the 25–29-year baseline per year).

	Sprint-F	Sprint-M	Endur-F	Endur-M
*Model comparison*				
R^2^ linear	0.874	0.819	0.944	0.918
R^2^ quadratic	0.984	0.954	0.990	0.987
R^2^ segmented	0.981	0.953	0.991	0.985
ΔAICc segmented vs. linear	25.1	15.4	21.7	20.3
ΔAICc segmented vs. quadratic	−1.02	−0.24	+1.18	−1.81
Stroke-specific fits preferring the segmented model (%)	50	50	—	—
Support (segmented vs. quadratic)	equivalent	equivalent	equivalent	equivalent
*Segmented model*				
Transition age (years)	62	67	67	57
95% CI (years)	57–62	62–67	62–72	52–62
Slope before (% · yr^−1^)	0.78	0.73	1.20	0.60
Slope after (% · yr^−1^)	3.37	3.66	2.92	2.01
Slope change (% · yr^−1^)	2.59	2.93	1.72	1.40
Ratio after/before	4.3	5.0	2.4	3.3
Davies’ test	*p* < 0.001	*p* < 0.001	*p* < 0.001	*p* < 0.001
*Quadratic model*				
Acceleration 2a_2_ (% · yr^−2^)	0.068	0.061	0.038	0.047

Sprint-F/Sprint-M: sprint events (50 and 100 m), female/male swimmers; Endur-F/Endur-M: endurance events (400 and 800 m), female/male swimmers. R^2^: coefficient of determination of each model fitted to the 13 age-group means (see Section 2.5.3); ΔAICc: AICc (linear or quadratic model) – AICc (segmented model), positive values favour the segmented model; |ΔAICc| < 2 indicates equivalent support. Transition age: breakpoint of the segmented model with its 95% confidence interval (CI), an approximate value at five-year resolution and not a precise physiological threshold; slopes: annual rate of deterioration before and after the transition age; slope change: post- minus pre-transition slope; ratio: post-/pre-transition slope; Davies’ test: test for the existence of a change in slope; 2a_2_: acceleration (second derivative) of the quadratic model.

## Data Availability

The datasets analysed are publicly archived as the official results of the Kyushu (Japan, 2023), Doha (Qatar, 2024) and Singapore (2025) World Aquatics Masters Championships on the official World Aquatics website (https://www.worldaquatics.com). The aggregated age-group dataset and the analysis code are available from the corresponding author upon reasonable request.
